# Antibacterial Activity, Probiotic Potential, and Biocontrol Efficacy of Two Lactic Acid Bacteria Against *Penicillium expansum* on Fresh Grapes

**DOI:** 10.3390/foods14030493

**Published:** 2025-02-04

**Authors:** Yuting Hou, Yaoke Duan, Guofang Wu, Jianbo Zhang, Xuan Luo, Miao Zhang, Huili Pang, Yuxuan Hao, Yanping Wang, Yimin Cai, Lei Wang, Zhongfang Tan

**Affiliations:** 1 Henan Key Laboratory of Ion-Beam Green Agriculture Bioengineering, School of Agricultural Sciences, Zhengzhou University, Zhengzhou 450001, Chinaduyk@nwafu.edu.cn (Y.D.); cai@affrc.go.jp (Y.C.); 2 Plateau Livestock Genetic Resources Protection and Innovative Utilization Key Laboratory of Qinghai Province, Key Laboratory of Animal Genetics and Breeding on Tibetan Plateau, Ministry of Agriculture and Rural Affairs, Academy of Animal Science and Veterinary Medicine, Qinghai University, Xining 810016, China

**Keywords:** lactic acid bacteria, probiotics, antimicrobial properties, biological control, grape

## Abstract

Lactic acid bacteria are commonly present in various sources and possess significant probiotic properties. They can inhibit pathogenic bacteria and fungi simultaneously, making them promising candidates as bio-preservatives. This study investigated two potential probiotic strains: *Lactiplantibacillus plantarum* LR5-2 (isolated from fermented meat products) and *Lacticaseibacillus rhamnosus* SQ63 (isolated from infant feces). The study evaluated their aggregation ability, anti-pathogenic activity, safety, and tolerance to gastrointestinal conditions, phenol, and bile salts. Additionally, their biological control potential against *Penicillium expansum* on fresh grapes was assessed. The results demonstrated that both strains exhibited high survival rates under extreme gastrointestinal conditions, enhanced Auto-aggregation, co-aggregation, and hydrophobicity. They displayed strong antioxidant activity and significant antibacterial effects against 11 pathogenic fungi and foodborne pathogens. Biosafety testing revealed that both strains are sensitive to most antibiotics, do not produce biogenic amines, and exhibit no hemolytic or DNase activity. In grapes, *L. plantarum* LR5-2 and *L. rhamnosus* SQ63 significantly reduced the incidence and disease index of *P. expansum* infection. In conclusion, the characterization analysis and bio-preservation experiments revealed that LR5-2 and SQ63 have strong potential as probiotics and bio-preservatives.

## 1. Introduction

Probiotics are live microorganisms that colonize the host’s gastrointestinal and reproductive systems. They exert beneficial effects by enhancing the balance of the host’s microbiota and supporting overall health. According to the Food and Agriculture Organization (FAO) and the World Health Organization (WHO), probiotics are defined as live microorganisms that, when administered in adequate amounts, confer health benefits to the host [1].

Probiotics regulate immune responses, facilitate the metabolism of toxic substances, and produce antimicrobial compounds, vitamins, and polysaccharides [2]. They also contribute to the restoration of the gut microbial balance by enhancing nutrient absorption [3,4]. Probiotic screening is typically based on several criteria, including strain source, safety, functional properties, and potential applications [5]. Lactic acid bacteria (LAB) and yeast are the most extensively studied and widely applied probiotic strains [6]. LAB are particularly favored for their safety, non-toxicity, non-pathogenic nature, and lack of carcinogenicity [7]. LAB can utilize milk, vegetables, fruits, and other nutrient-rich substances as their nutritional foundation [8,9] and can improve the intestinal tract and enhance the body’s immunity.

LAB, which are widely used in the food industry, healthcare sector, and feed and cosmetic industries, are recognized as Generally Recognized as Safe (GRAS) organisms [10]. LAB can colonize the intestine after being ingested by the human body. They resist the adhesion and colonization of harmful bacteria, inhibit the growth and reproduction of these bacteria, and reduce the production of endotoxins [11]. LAB also enhance the intestinal barrier, regulate the composition and activity of intestinal flora, prevent and treat diarrhea and peptic ulcers, reduce serum cholesterol, improve human immunity, and contribute to anti-aging [12]. Furthermore, LAB have long been used for food preservation, and their history can be traced back to the origin of agriculture. They play a key role in combating bacterial infections and maintaining the stability of the gastrointestinal ecosystem by inhibiting various pathogens through mechanisms such as competitive exclusion (competing for nutrients and/or space) and the production of antimicrobial metabolites, including lactic acid, acetic acid, hydrogen peroxide, carbon dioxide, diacetyl, and bacteriocins [13]. It has been reported that bacteriocins produced by *Pediococcus* have a better preservation effect on strawberries, tomatoes, corn, and mushrooms than sodium benzoate and sulfites [14]. *Limosilactobacillus fermentum* inhibits the growth of *Aspergillus chevalieri*, thereby extending the shelf life of bread by 30 days [15]. Both *Lactococcus lactis* subsp. [16] and *Lactobacillus casei* [17] effectively inhibit the growth of foodborne pathogens in dairy products. Using LAB and sodium alginate, Li et al. [18] created an edible coating that extended the shelf life of strawberries and reduced microbial growth during storage.

Grapes (*Vitis vinifera* L.), known for their unique flavor and high nutritional value, are among the oldest cultivated fruits in the world [19]. However, fresh grapes are highly susceptible to infection by a variety of fungal pathogens, particularly by *Penicillium* spp., leading to significant postharvest decay losses [20]. It has been estimated that 10–40% of total grape production is lost due to this postharvest fungal decay. The fungal rot develops majorly because of infections by latent and quiescent fungi or infections of berries through wounds during harvest and transport [21]. Currently, sulfur dioxide (SO_2_) fumigation is the most commonly used method to prevent grape fungal contamination [22]. However, sulfur dioxide is not only phytotoxic but may also cause sulfite intolerance in some consumers [23]. Therefore, considering the safety, antimicrobial properties, and ability of LAB to colonize vulnerable plant tissues, LAB represent ideal candidates for managing postharvest fungal decay.

The human intestinal tract and fermented foods are the two main sources of LAB [24,25]. LAB derived from the human intestinal tract are relatively safe, while those from fermented foods exhibit greater diversity. These two sources represent key research areas in the study of LAB. When SO_2_ fumigation is used to preserve the quality of postharvest table grapes, controlling the residual levels remains challenging. Excessive SO_2_ residues resulting from fumigation may not only pose health risks to humans but also negatively impact the flavor of the grapes. Therefore, the development of natural, residue-free, and non-harmful biological preservatives as alternatives to traditional chemical preservatives has become an important research focus in grape preservation. This study aims to screen LAB with broad-spectrum antibacterial proprieties from the human intestinal tract and fermented meat products, evaluate their potential probiotic characteristics and safety, and apply them to the biological control of *P. expansum* on fresh grapes. The goal is to promote the application of lactic acid bacteria in biological preservation and functional food as a healthy alternative to sulfur dioxide.

## 2. Materials and Methods

### 2.1. Isolation, Screening, and Identification of LAB

#### 2.1.1. Isolation of LAB

Fecal samples of healthy elderly individuals and infants were collected from Zhengzhou and Xinxiang, Henan Province. Bacon and sausage samples were obtained from Hubei Province, and milk tablets were purchased from local supermarkets in Zhengzhou. The samples were serially diluted with sterile saline, and appropriate dilutions were added onto De Man, Rogosa, and Sharpe (MRS) agar plates. The plates were incubated at 37 °C for 48 h in an anaerobic workstation (DG250, Don Whitley Scientific, Bingley, UK). The colonies were examined for their morphology, and strains that were round, milky-white, smooth-surfaced, and relatively moist were selected from the agar plates. The isolated and purified strain was placed into an NB medium containing 10% dimethyl sulfoxide (DMSO) in cryovials and stored at −80 °C.

#### 2.1.2. Screening of LAB

*Escherichia coli* ATCC 11775 and *Salmonella typhimurium* ATCC 13076 were used as indicator bacteria. The agar well diffusion method was employed for antibacterial assessment [26]. The pathogens (1 × 10^10^ CFU/mL) were evenly mixed into nutrient agar. A 10 mm hole punch was used to create wells in the agar plates, and 200 μL of LAB cell-free supernatant was added to each well. The plates were incubated at 37 °C for 24 h, and the diameter of the inhibition zone was measured.

*Aspergillus flavus* BNCC 142803 and *P. expansum* BNCC 14644 were used as fungal indicator organisms to screen for strains with broad-spectrum antibacterial ability using the double-layer plate method [27]. The lower layer consisted of 20 mL of MRS agar, which was allowed to solidify before two 3 cm streaks of LAB were applied and incubated for 1 h. The upper layer had 5 mL of PDA containing 3% pathogenic fungi (1 × 10^6^ CFU/mL) poured on it and was incubated for 5–7 d to assess antibacterial activity.

Two strains, LR5-2 and SQ63, demonstrating broad-spectrum antibacterial activity against bacteria and fungi, were identified.

#### 2.1.3. Molecular Identification of LAB

The strains LR5-2 and SQ63 were identified through 16S rDNA sequencing performed by BGI Genomics Institute (Beijing, China). Total genomic DNA was extracted [28], and PCR (polymerase chain reaction) was performed using universal primers (27F: 5′AGAGTTTGATCCTGGCTCAG-3′ and 1492R: 5′-TACGGYTACCTTGTTACGACTT-3′). The resulting product was sequenced in the 16S rDNA gene region, and the isolates were identified through sequence alignment using the NCBI BLAST platform. The query coverage and percent identity of LR5-2 are both 100%, while the query coverage and percent identity of SQ63 are 99% and 99.12%, respectively. These values significantly surpass those of other bacterial species in terms of sequence coverage and similarity.

### 2.2. Assessment of Anti-Pathogenicity and Aggregation Ability

#### 2.2.1. Anti-Pathogenicity

The antibacterial activity of the screened LAB was tested against *E. coli* ATCC 11775, *Staphylococcus aureus* ATCC 6538, *Pseudomonas aeruginosa* ATCC 15692, *Micrococcus luteus* ATCC 4698, *Listeria monocytogenes* ATCC 51719, *S. typhimurium* ATCC 13076, *A. flavus* BNCC142803, *P. expansum* BNCC 14644, *Aspergillus niger*, *Penicillium citrinum*, and *Penicillium georgiense* using the method described in Section 2.1.2. All these strains are preserved in the Henan Key Laboratory of Ion-Beam Green Agriculture Bioengineering.

#### 2.2.2. Automatic Aggregation Ability

The probiotic strains LR5-2 and SQ63 were cultured in MRS broth for 36 h and centrifuged at 4 °C and 8000 rpm using an Eppendorf 5810/R centrifuge (Eppendorf AG, Hamburg, Germany) for 10 min. The precipitates were washed twice with phosphate-buffered saline (PBS) and re-suspended in PBS. UV absorbance measurements were performed using a UVmini-1240 spectrophotometer (Shimadzu Corporation, Kyoto, Japan). The cell suspension was adjusted to an OD600 of approximately 0.8 and incubated at 37 °C for 24 h. At 0, 2, 4, 6, 12, and 24 h, culture samples were taken to determine the absorbance at OD600. Each test was repeated three times, and the Auto-aggregation [29] ability was calculated according to Formula (1):

Auto-aggregation rate (%) = (1 − A_1_/A_0_) × 100% (1)

where A_1_ represents the absorption value of culture medium at time T, and A_0_ represents the initial absorbance value of the bacteria.

#### 2.2.3. Collaborative Aggregation Capability

The co-aggregation ability of the isolated strains was determined using the method described by Divyashree et al. [30] and Wang et al. [31], with minor modifications. The cell suspension was prepared as described in Section 2.2.2. LAB were mixed with an equal volume of pathogenic bacteria (*S. typhimurium*, *E. coli*, *L. monocytogenes*, *S. aureus*, *P. aeruginosa*) at a concentration of 1 × 10^8^ CFU/mL and incubated at 37 °C for 24 h. A control test tube containing only LAB and pathogenic strains was included. Absorbance values were measured at 0, 5, and 24 h. The co-aggregation ability was calculated using Formula (2):

Co-aggregation rate (%) = [1 − (A_m_/(A_a_ + A_b_)/2)] × 100% (2)

where A_m_ is the absorption value after mixing, A_a_ is the absorbance value of LAB, and A_b_ is the absorbance value of pathogenic bacteria.

### 2.3. Cell Surface Hydrophobicity

The cell suspension was prepared according to Section 2.2.2. The cell suspension (3 mL) was vortexed (LC-Vortex MF, Shanghai, China) for 60 s at 3000 rpm with 2 mL of either xylene or ethyl acetate and incubated at 37 °C for 1 h. The organic and aqueous phases were separated. Finally, the absorbance of the aqueous phase was measured at 600 nm and calculated according to Formula (1).

### 2.4. Evaluation of Environmental Tolerance

#### 2.4.1. Emergency Tolerance: Stability to Simulated Gastrointestinal Fluid

The simulation of the digestion stages of the two probiotics in the mouth, stomach, and small intestine was conducted according to the method described by Guo et al. [32]. To each 20 mL of LR5-2 and SQ63 (OD600 of approximately of 0.8), 20 mL of the following was added: salivary α-amylase solution (2 g/L, pH 7.0), pepsin solution (3 g/L, pH 2.5), trypsin (10 g/L, pH 7.0), and bile salt solution (3 g/L, pH 7.0). For the first 0–1 h, each strain underwent oral digestion simulation. After 1–5 h, the strain was in the simulated gastric digestion solution, and from 5 to 11 h, it underwent simulated intestinal digestion solution. At each time point, an appropriate amount of digest was sampled and subjected to dilution coating count using a concentration gradient dilution method. A mixture of LAB suspension and sterile water served as a negative control, and the remaining operations were the same as in the experimental group. All treatments were repeated three times.

#### 2.4.2. Bile Salt Tolerance

The prepared cell suspension was inoculated into a liquid MRS medium containing 0.3% (*w*/*v*) bovine bile salt at an inoculation rate of 0.3%. Gradient dilution plating was performed after 1, 2, 3, and 4 h of incubation. The bile salt tolerance of LAB was assessed by determining the number of viable bacteria on the plates [33].

#### 2.4.3. Phenol Resistance

The cell suspension was inoculated into MRS broth containing 0.3%, 0.5%, and 0.7% (*w*/*v*) phenol and incubated at 37 °C for 24 h. After diluting the samples with sterile PBS buffer, the survival rate at each phenol concentration was determined by counting the number of viable cells.

### 2.5. Antioxidant Activity

#### 2.5.1. DPPH Radical Scavenging Activity

The 2,2-diphenyl-1-picrylhydrazyl (DPPH) free radical scavenging activity of the strain was assessed with slight modifications to the method described by Méndez-Galarraga et al. [34]. The strains were mixed with 2 mM DPPH–methanol solution and incubated in the dark at 37 °C for 30 min. The control group consisted of PBS and DPPH–methanol solution, while the blank group included the strain and methanol. After centrifugation at 8000 rpm for 10 min, the supernatant was measured in triplicate at 517 nm using a Varioskan LUX microplate reader (Thermo Fisher Scientific, Waltham, MA, USA). DPPH scavenging activity (%) was calculated using Formula (3).

Scavenging activity (%) = [1 − (A_sample_ − A_blank_)/control] × 100% (3)

where A_sample_ is the absorbance of the sample, A_blank_ is the absorbance of the blank group, and control is the absorbance of the control group.

#### 2.5.2. ABTS Radical Scavenging Activity

The 2,2′-azobis (3-ethylbenzothiazoline-6-sulfonate) (ABTS) free radical scavenging activity of each strain was evaluated according to the method described by Sheng et al. [35]. The ABTS reaction solution was prepared by mixing a 7 mM ABTS solution with 2.45 mM potassium persulfate and storing it the dark at room temperature for 12−16 h. The reaction solution was diluted to an absorbance of 0.7 ± 0.02 at 734 nm for use as the test solution. For the assay, 100 μL of extract was added to 3 mL of ABTS reaction solution. The control group consisted of PBS and ABTS, while the blank group included the strain sample and PBS. After incubation in the dark for 6 min, absorbance was measured at 734 nm. The calculation method was the same as that used for DPPH free radical scavenging activity.

### 2.6. Safety Evaluation

#### 2.6.1. DNase Activity and Hemolytic Activity

The ability of the isolated strains to produce DNase was evaluated using the method of Boricha et al. [36]. The two isolated LAB strains were streaked on DNase agar and incubated at 37 °C for 48 h. The presence of a clear zone around the colony indicated DNase activity, and colonies with a distinct pink zone were considered DNase-positive.

For hemolysis testing, overnight cultures of the isolated LAB were streaked onto 5% (*w*/*v*) Colombian blood agar plates. *S. aureus* served as a positive control. Plates were incubated at 37 °C for 48 h, and hemolysis was assessed as follows: β-hemolysis (transparent zone around the colony) indicated positive hemolysis, while α-hemolysis (a green zone) and γ-hemolysis (no zone) were classified as being negative for hemolysis [37].

#### 2.6.2. Production of Biogenic Amines

The ability of the isolated strains to produce biogenic amines was evaluated according to the method of Martín et al. [38]. Each LAB isolate was passaged five times in MRS broth containing 0.1% (*w*/*v*) of each precursor amino acid. Pyridoxal-5-phosphate (0.005%) was added to the MRS broth to promote enzyme induction. The isolates were streaked in triplicate onto MRS agar containing each amino acid and incubated at 37 °C for 48 h. The plate without precursor amino acids served as the negative control. A positive result was indicated by the disappearance of purple coloration or tyrosine precipitation around the colony.

#### 2.6.3. Antibiotic Sensitivity

The sensitivity of two LAB strains to several commonly used and clinically significant antibiotics was determined using the agar disk diffusion method [39]. The antibiotics included erythromycin (15 μg), penicillin (1 μg), tetracycline (30 μg), chloramphenicol (10 μg), streptomycin (10 μg), rifampicin (5 μg), vancomycin (30 μg), kanamycin (30 μg), gentamicin (10 μg), doxycycline (30 μg), and ampicillin (30 μg). A 100 μL cell suspension (1 × 10^9^ CFU/mL) was evenly spread on MRS agar and antibiotic disks were placed on the surface. After incubation at 37 °C for 24 h, the diameter of the inhibition zones around the antibiotic disks was measured using a vernier caliper, with precision up to two decimal places (0.01 mm). Each test was repeated three times.

### 2.7. Probiotic Strains for the Biological Control of P. expansum on Fresh Grapes

The biocontrol of grape *P. expansum* was conducted using the method of Zhang et al. [40] with appropriate modifications. LR5-2 and SQ63 were cultured in MRS broth for 36 h, and the concentration of the fermentation broth was adjusted to 1 × 10^10^ CFU/mL. The fermentation broth was divided into four groups: (1) fermentation broth without probiotics, (2) fermentation broth of *L. plantarum* LR5-2, (3) fermentation broth of *L. rhamnosus* SQ63, and (4) fermentation broth of *L. plantarum* LR5-2 + *L. rhamnosus* SQ63. Grapes were soaked in each treatment group for approximately 2 min and allowed to air dry in an ultra-clean bench. A disposable needle was used to make a 1 cm deep puncture in the surface of each fruit, and 10 μL of *P. expansum* spore suspension (1 × 10^6^ CFU/mL) was inoculated into each wound. After inoculation, the fruits were stored at 25 °C. The incidence of disease was calculated after 5–7 days of storage as the percentage of infected fruits out of the total number of fruits. Twenty fruits were combined as one biological replicate, with three biological replicates for each treatment.

### 2.8. Statistical Analysis

All experiments contained three replicates, and the data are presented as mean ± standard deviation. One-way ANOVA was conducted using IBM SPSS Statistics (IBM Corp., Armonk, NY, USA), and Duncan post hoc tests were used to compare the means, with *p* < 0.05 deemed statistically significant.

## 3. Results and Discussion

### 3.1. Strain Identification

**Figure 1:**
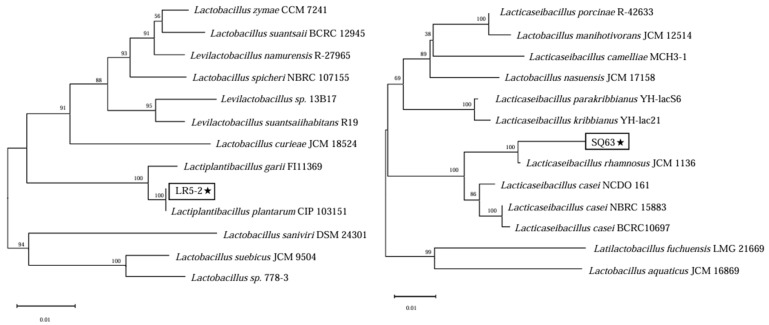
The phylogenetic tree of evolutionary relationship of two isolated LAB, with the strains marked with an asterisk (★). The bar indicates the number of nucleotide substitutions per site and percentages are indicated at the nodes with bootstrapping values for 1000% replicates of data.

In this study, the 16S rDNA gene sequences of strains LR5-2 and SQ63 were sequenced and compared to known isolates in the NCBI BLAST database. The sequence alignment results are shown in Figure 1. Phylogenetic analysis confirmed that strain LR5-2, isolated from bacon, was *L. plantarum*, while strain SQ63, isolated from infant feces, was *L. rhamnosus*.

### 3.2. Auto-Aggregation, Co-Aggregation, and Hydrophobicity Analyses of Probiotic Strains

**Figure 2:**
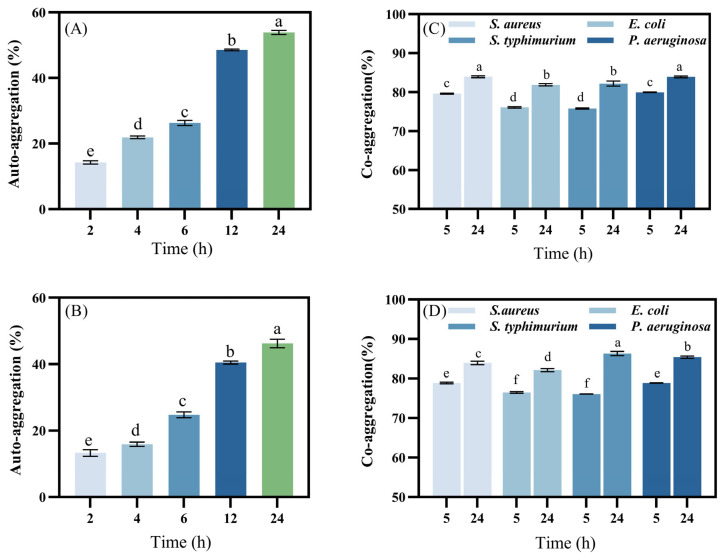
Auto-aggregation and Co-aggregation ability of isolates. The Auto-aggregation ability (**A**) and co-aggregation ability (**B**) of *L. plantarum* LR5-2. The Auto-aggregation ability (**C**) and co-aggregation ability (**D**) of *L. rhamnosus* SQ63. The different lowercase letters indicate significant differences between the mean values compared (*p* < 0.05). The same is below.

The adhesion ability of LAB reflects their capacity to colonize the small intestinal wall and interact with intestinal epithelial cells. Hydrophobicity and Auto-aggregation are key factors that partially represent this adhesion capability. Strains with high Auto-aggregation ability are better positioned to compete for cell matrix–host binding sites [41]. Additionally, these strains can prevent pathogens from binding to the host, thereby inhibiting the adhesion of pathogenic bacteria. Figure 2A,C illustrates the Auto-aggregation ability of *L. plantarum* LR5-2 and *L. rhamnosus* SQ63, respectively. The Auto-aggregation rates of both strains increased over time, rapidly agglomerating within 12 h and reaching their highest values by the end of the test. At 24 h, the Auto-aggregation rates of *L. plantarum* LR5-2 and *L. rhamnosus* SQ63 were 53.85 ± 0.64% and 46.22 ± 1.32%, respectively. These results demonstrate high Auto-aggregation ability [29], highlighting their strong potential to adhere to intestinal epithelial cells.

The co-aggregation ability of LAB reflects their capacity to adhere to specific strains. This characteristic plays a crucial role in eliminating the colonization of pathogens by preventing them from attaching to host tissues [31]. Figure 2B,D illustrates the co-aggregation ability of *L. plantarum* LR5-2 and *L. rhamnosus* SQ63, respectively. The data show that both strains achieved high co-aggregation levels within 5 h for *E. coli*, *S. aureus*, *P. aeruginosa*, and *S. typhimurium*, with co-aggregation rates exceeding 75%. After 24 h, the co-aggregation ability increased to more than 80%. This is consistent with the findings of Divyashree et al. [30], confirming that the two strains can quickly adhere to various pathogens, thereby inhibiting their attachment in the host tissues in the gastrointestinal tract. Furthermore, these strains produce antimicrobial compounds that significantly reduce or eliminate harmful microorganisms [42].

**Figure 3:**
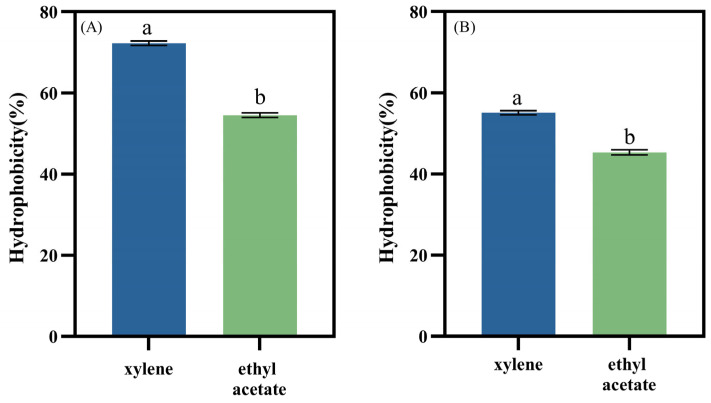
Hydrophobicity of *L. plantarum* LR5-2 (**A**) and *L. rhamnosus* SQ63 (**B**). The different lowercase letters indicate significant differences between the mean values compared (*p* < 0.05).

LAB enhance their adhesion in the intestinal tract through strong hydrophobic interactions with intestinal epithelial cells. High hydrophobicity contributes to better intestinal adhesion, reducing the likelihood of expulsion due to intestinal peristalsis [3]. This characteristic is a significant indicator for evaluating adhesion potential. Hernández-Alcántara et al. [43] classify the hydrophobicity of strains into three levels: high (≥60%), moderate (40–60%), and low (≤40%). The hydrophobicity of the isolated strains towards xylene and ethyl acetate is presented in Figure 3. Both LAB strains exhibited significantly higher hydrophobicity toward xylene than ethyl acetate. Specifically, *L. plantarum* LR5-2 showed hydrophobicity values of 72.22 ± 0.55% for xylene and 54.51 ± 0.56% for ethyl acetate, indicating high hydrophobicity. *L. rhamnosus* SQ63 displayed hydrophobicity values of 58.59 ± 0.51% for xylene and 45.32 ± 0.62% for ethyl acetate, reflecting moderate hydrophobicity. These results align with previous findings [44,45], which reported that certain LAB strains exhibit a stronger affinity for non-polar acidic solvents like xylene while showing lower affinity for ethyl acetate, a non-polar basic solvent [46].

### 3.3. Broad-Spectrum Antibacterial Activity of Isolated Strains

Inhibiting the growth of harmful and pathogenic bacteria is a key function of certain probiotics. For example, LAB secrete specific metabolites that disrupt the cell structure or alter the living environment of pathogens. This mechanism enables LAB to effectively compete with or inhibit pathogenic bacteria, reducing the toxins secreted by harmful microbes and promoting the dominance of beneficial bacteria in the gastrointestinal tract [47]. The antibacterial activity of *L. plantarum* LR5-2 and *L. rhamnosus* SQ63 against Gram-positive and Gram-negative bacteria is presented in Table 1. Both isolates demonstrated significant inhibitory effects on pathogenic bacteria, with the strongest inhibition observed against *M. luteus*, measuring 25.97 ± 0.27 mm and 26.06 ± 0.45 mm, respectively. Furthermore, the inhibitory effect against Gram-positive bacteria was stronger than that against Gram-negative bacteria. For Gram-positive bacteria, the inhibition zones exceeded 20 mm, while for Gram-negative bacteria, the zones were greater than 15 mm. These findings align with the results of Assari et al. [48] and Hashemi et al. [49], which demonstrate that *L. plantarum*, *Lacticaseibacillus casei*, and *Lactobacillus acidophilus* exert stronger inhibitory effects on Gram-positive bacteria than on Gram-negative bacteria. The likely explanation is that LAB secrete lactic acid and antimicrobial peptides, which lower the pH of the environment, disrupt the cell structure of Gram-positive bacteria, and inhibit their growth. In contrast, Gram-negative bacteria possess an outer membrane rich in lipopolysaccharides, which provides a physical barrier that mitigates the effects of the metabolic products of LAB. However, other studies have reported that the bacteriocins produced by LAB exhibit stronger antibacterial activity against Gram-negative bacteria [50].

Probiotics show significant potential for applications across various fields, including food science, agriculture, and the medical industry. However, these areas, especially the food sector, are highly susceptible to mold contamination. As biological preservatives, LAB produce a variety of antifungal compounds, effectively inhibiting fungal contamination in food products. Additionally, some LAB strains reduce fungal toxins in the environment through adsorption or biodegradation [51]. The antibacterial activity of *L. plantarum* LR5-2 and *L. rhamnosus* SQ63 against several common pathogenic fungi is shown in Table 2. The results indicate that both LR5-2 and SQ63 exhibit the strongest inhibitory effects against *A. flavus*, *P. expansum*, *P. georgia,* and *P. citrinum* and a moderate inhibitory effect against *A. niger*. The potent antifungal activity of LAB is attributed to the production of various metabolites, including bacteriocins, organic acids (e.g., lactic acid, acetic acid, and propionic acid), ethanol, diacetyl, and hydrogen peroxide [52,53]. This study highlights the significant antagonistic effects of *L. plantarum* LR5-2 and *L. rhamnosus* SQ63 against common pathogenic bacteria responsible for foodborne poisoning, including *E. coli*, *L. monocytogenes*, *S. typhimurium*, and *S. aureus*. Additionally, these strains exhibit strong inhibitory activity against common mycotoxigenic and spoilage fungi such as *Aspergillus* spp. and *Penicillium* spp., which are frequently encountered during food processing and storage. Many researchers have found that LAB exhibit strong inhibitory effects against *Colletotrichum gloeosporioides*, *Botryodiplodia theobromae*, *Penicillium digitatum*, and several foodborne pathogens. Through soaking or in vivo antagonistic assays, the shelf life of mangoes [54], citrus fruits [55], apples, and sugarcane juice has been extended [56]. Grape skin is thin and has a relatively low pH, rendering it highly susceptible to fungal infection and colonization. However, *L. plantarum* LR5-2 and *L. rhamnosus* SO63 demonstrate potent antifungal activity, indicating that soaking treatments or in vivo antagonistic approaches could be utilized for grape preservation. These strategies show considerable promise as biological preservatives.

### 3.4. Survival of Probiotics Under Simulated Oral and Gastrointestinal Conditions

**Figure 4:**
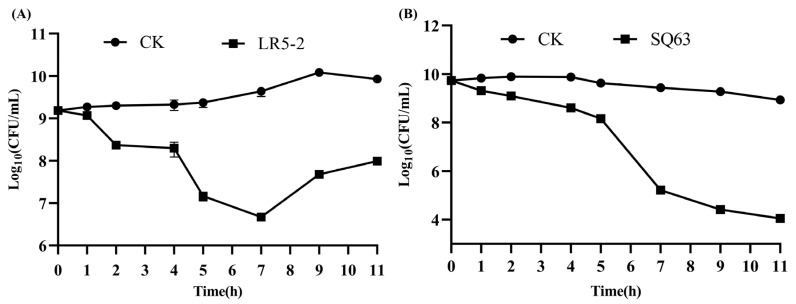
Total bacterial counts of *L. plantarum* LR5-2 (**A**) and *L. rhamnosus* SQ63 (**B**) in simulated oral and gastrointestinal environments.

High survival rates of probiotic strains within the gastrointestinal tract are essential for their beneficial effects, as most strains cannot survive its harsh environment [57]. Simulated gastrointestinal fluid tolerance serves as a key indicator for evaluating probiotic potential. The survival of the isolated strains under simulated oral and gastrointestinal conditions is shown in Figure 4. During the in vitro simulated oral digestion process, the colony counts of *L. plantarum* LR5-2 and *L. rhamnosus* SQ63 showed a slight decrease, suggesting that α-amylase had minimal effect on these strains. In the subsequent simulated gastric digestion stage, their colony counts decreased to 8.28 ± 0.15 and 8.62 ± 0.06 Log10 CFU/mL, respectively. Overall, the colony counts of both strains decreased after oral and gastric digestion, indicating that exposure to acidic conditions and digestive enzymes likely compromised the permeability of the cell membranes. Some researchers [58,59] have suggested that cells capable of surviving for 2–3 h in a simulated gastric environment can endure the entire digestive process in the stomach. After 5 h, the strains entered the simulated intestinal digestion stage. *L. plantarum* LR5-2 initially exhibited a decrease in colony count, followed by an increase, suggesting an adaptation period to the intestinal environment. This result is similar to that of Dos Santos et al. [60] and may be linked to the higher survival rate of *L. plantarum* LR5-2 during the first two hours in the presence of bile salts. Some studies suggest that most LABs can tolerate bile salts in simulated intestinal fluid [33,60]. Furthermore, this increase may be attributed to the bile salt dissociation ability of the test strains and the relatively mild pH in the environment [61]. In contrast, *L. rhamnosus* SQ63 displayed a consistent decline in colony count, with a final colony count of 4.04 ± 0.09 Log10 CFU/mL. This may indicate that SQ63 is more sensitive to pancreatic enzymes in the presence of bile salts [62]. Previous studies have demonstrated that resistance to bile salts and pancreatic enzymes can vary among different species and strains [63]. Based on these findings, *L. plantarum* LR5-2 and *L. rhamnosus* SQ63 show potential as probiotic strains. This study provides preliminary data supporting the gastrointestinal tolerance of lactic acid bacteria. However, due to the absence of in vivo experiments, further validation of their survival and colonization capabilities in the gastrointestinal tract is necessary through animal or clinical trials. Future research should incorporate in vivo studies to further assess the tolerance and potential clinical applications of various lactic acid bacteria within the gastrointestinal system.

### 3.5. Bile Salt, Phenol Tolerance, and Antioxidant Capacity

**Figure 5:**
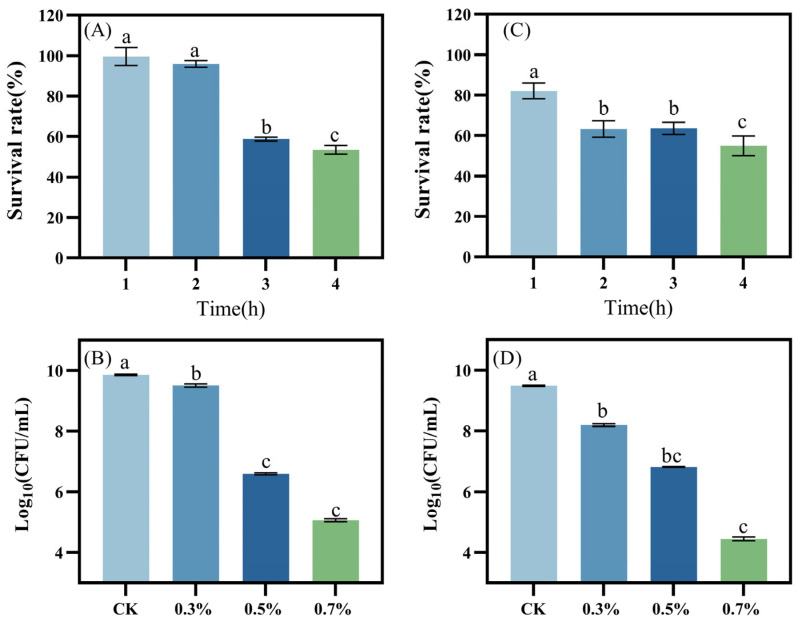
Tolerance results of isolated strains. The survival rate of *L. plantarum* LR5-2 under 0.3% (*w*/*v*) bile salt conditions (**A**) and tolerance at different phenol concentrations (**C**). The survival rate of *L. rhamnosus* SQ63 under 0.3% (*w*/*v*) bile salt conditions (**B**) and the tolerance under different phenol concentrations (**D**). The different lowercase letters indicate significant differences between the mean values compared (*p* < 0.05).

Bile salt, formed from bile acid and sodium or potassium salts, promotes the digestion and absorption of lipids [64]. Upon entering the small intestine, probiotics encounter high concentrations of bile salts, which impact their colonization and reduce their efficacy [65]. The survival rate of the isolated strains in a medium containing 0.3% bile salt over 4 h is shown in Figure 5A,B. *L. plantarum* LR5-2 exhibited robust survival during the initial 2 h, with only a minor decline, and a survival rate reaching 95.92 ± 1.70%. A significant reduction was observed at 3 h, but the survival rate remained at 53.41 ± 2.16% at the end of the experiment. Similarly, *L. rhamnosus* SQ63 (Figure 5B) experienced a moderate decline in the first 2 h, followed by stabilization. At the end of the experiment, its survival rate was 54.92 ± 4.93%. The differences in bile salt tolerance among LAB strains may be attributed to variations in bile salt hydrolase content and other strain-specific characteristics [66].

Probiotics must exhibit resistance to phenol to survive in the host gastrointestinal environment. Intestinal bacteria can deaminate amino groups in food proteins, converting them into aromatic amino acids, which subsequently produce phenols. Although phenol concentrations in the intestine are low, they can still affect the survival of probiotics. Notably, some LAB strains lack phenol tolerance [67]. The tolerance of the isolated strains to phenol is illustrated in Figure 5C,D. As phenol concentrations increased, the viable counts of *L. plantarum* LR5-2 and *L. rhamnosus* SQ63 decreased compared to the control group. At a phenol concentration of 0.3% (*w*/*v*), the viable counts for these two strains were 9.51 ± 0.05 and 8.19 ± 0.04 Log10 CFU/mL, respectively. At a higher concentration of 0.7% (*w*/*v*), the viable counts reached their lowest levels, measuring 5.07 ± 0.04 and 4.45 ± 0.05 Log10 CFU/mL, respectively. Both strains demonstrated strong to moderate survival in a 0.5% bile salt environment, further highlighting their ability to withstand adverse conditions. Furthermore, these bacteria can survive by metabolizing toxic compounds like phenol, which are produced through the deamination of certain amino acids by gut microbiota [68]. These findings suggest that *L. plantarum* LR5-2 and *L. rhamnosus* SQ63 are promising probiotic candidates with the ability to resist the toxic effects of phenol.

**Figure 6:**
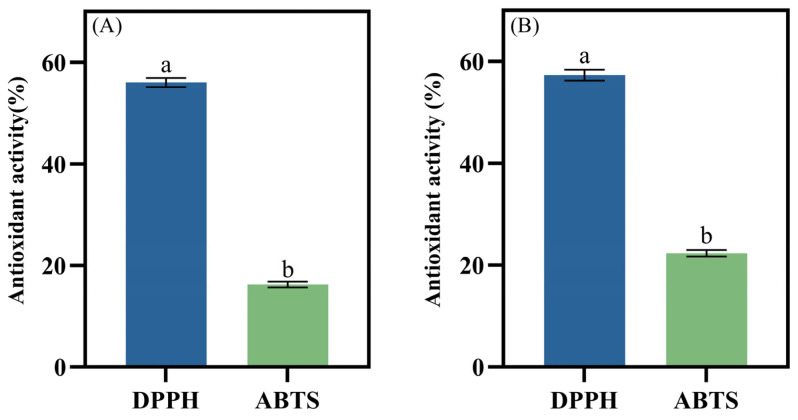
Antioxidant activity of *L. plantarum* LR5-2 (**A**) and *L. rhamnosus* SQ63 (**B**). The different lowercase letters indicate significant differences between the mean values compared (*p* < 0.05).

The antioxidant activity of potential probiotic strains was assessed using DPPH and ABTS free radical scavenging assays. Previous studies suggest that enhanced antioxidant capacity in the body may play a role in the prevention of certain chronic diseases, delay aging, and mitigate oxidative damage [69]. LAB are regarded as high-quality natural antioxidants, and their consumption can effectively counteract oxidative stress. An essential indicator of a strain’s tolerance to oxidative stress is its ability to scavenge DPPH free radicals, a key measure of antioxidant activity. The results of the antioxidant assays for the isolated strains are presented in Figure 6. *L. plantarum* LR5-2 exhibited a DPPH free radical scavenging rate of 56.02 ± 0.93% and an ABTS free radical scavenging rate of 16.25 ± 0.57%. The DPPH and ABTS free radical scavenging rates of *L. rhamnosus* SQ63 were 57.28 ± 1.06% and 22.32 ± 0.63%, respectively. The observed antioxidant activity of the LAB strains is attributed to their production of exopolysaccharides, bioactive peptides, and antioxidant enzymes [70]. Moreover, LAB contribute to maintaining intestinal microflora homeostasis through their antioxidant activity [35].

### 3.6. Safety Evaluation of Isolated Strains

#### 3.6.1. Hemolytic Activity and Biogenic Amine Production

**Figure 7:**
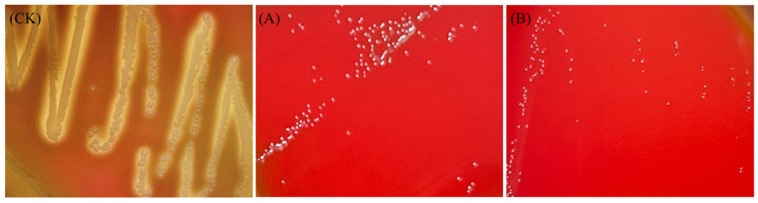
Hemolytic activity of *S. aureus* (CK), *L. plantarum* LR5-2 (**A**), and *L. rhamnosus* SQ63 (**B**).

Some strains produce hemolysin, an enzyme that lyses red blood cells, potentially causing internal defects, antigen–antibody reactions, anemia, and other health issues [71]. Therefore, hemolytic activity is a critical safety indicator when evaluating LAB. The hemolytic activity of *L. plantarum* LR5-2 and *L. rhamnosus* SQ63 is presented in Figure 7. A positive result was confirmed for CK (*S. aureus*), as clear zones were observed around its colonies. In contrast, both *L. plantarum* LR5-2 and *L. rhamnosus* SQ63 formed milky white colonies without visible hemolytic zones, indicating negative results. These findings suggest that *L. plantarum* LR5-2 and *L. rhamnosus* SQ63 do not exhibit hemolytic activity and are unlikely to cause damage to blood cells.

Biogenic amines are nitrogen-containing organic compounds that are produced during metabolism and are primarily formed through the decarboxylation of amino acids such as histidine, tyrosine, arginine, lysine, and ornithine by amino acid decarboxylases produced by LAB [72]. These compounds, including histamine, tyramine, spermine, cadaverine, and putrescine, are commonly found in fermented foods. Excessive accumulation of biogenic amines in the body can lead to severe reactions, such as headaches, respiratory disturbances, palpitations, and vomiting [73]. The two bacterial strains in this study did not produce biogenic amines or exhibit DNase activity, suggesting the absence of amino acid decarboxylase and their inability to generate harmful amine compounds.

#### 3.6.2. Antibiotic Sensitivity Evaluation

In clinical treatment, the widespread use of antibiotics has led to strains gradually developing strong drug resistance. Long-term consumption of drug-resistant LAB may pose risks to treatment [74]. In this study, the antibiotic tolerance of LAB was assessed using the agar disk diffusion method (K-B method). The sensitivity of the two isolates to 11 antibiotics is summarized in Table 3. Both *L. plantarum* LR5-2 and *L. rhamnosus* SQ63 were sensitive to most antibiotics but exhibited resistance to streptomycin, vancomycin, and kanamycin. Additionally, *L. plantarum* LR5-2 showed moderate sensitivity to tetracycline, while *L. rhamnosus* SQ63 was resistant to ampicillin. Studies have shown that several LAB strains possess intrinsic resistance to streptomycin and vancomycin, resistance that is typically not transferable to pathogenic bacteria [75]. Furthermore, certain lactobacilli isolated from fermented milk exhibit atypical resistance to β-lactam antibiotics [76]. The inherent resistance to vancomycin, a cell wall synthesis inhibitor, is primarily attributed to the presence of D-alanine, associated with D-alanine ligase [77,78]. Based on these findings, the LAB strains in this study exhibit sensitivity to most antibiotics and demonstrate adequate safety profiles.

### 3.7. Effects of LR5-2 and SQ63 on P. expansum Infection in Grapes

**Figure 8:**
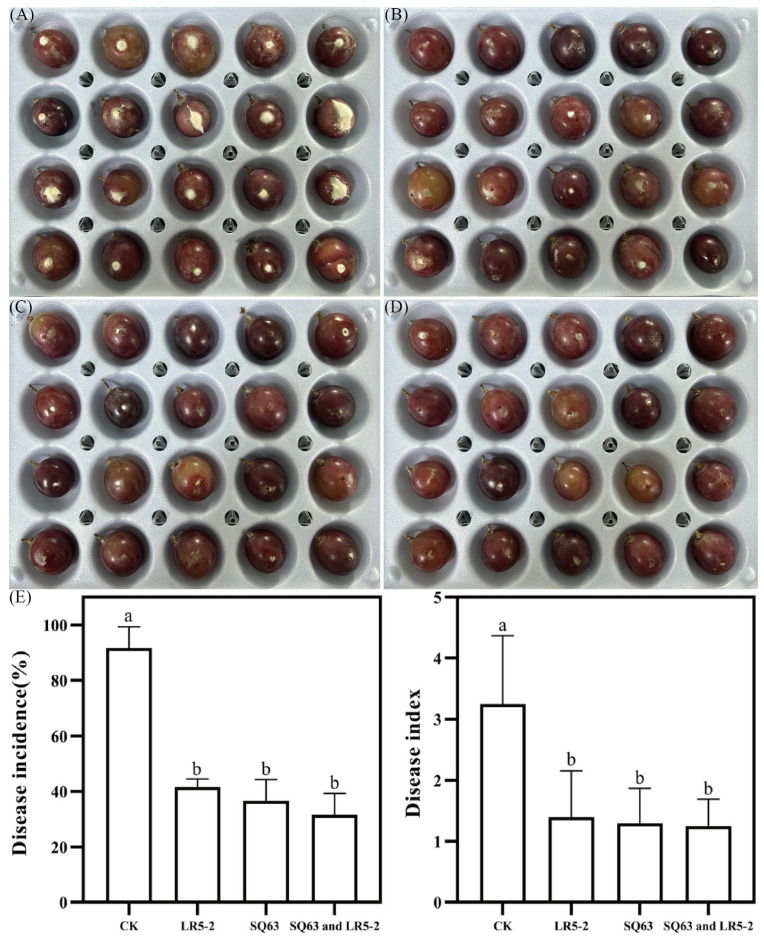
Effects of isolated strains on biological control of *P. expansum* in table grapes. (**A**) Fermentation broth without probiotics, (**B**) *L. plantarum* LR5-2 fermentation broth, (**C**) *L. rhamnosus* SQ63 fermentation broth, (**D**) *L. plantarum* LR5-2 + *L. rhamnosus* SQ63 fermentation broth, and (**E**) disease incidence and disease index of grape. The different lowercase letters indicate significant differences between the mean values compared (*p* < 0.05).

*Aspergillus* and *Penicillium* are among the most common fungal genera responsible for contaminating fruits and vegetables during storage. Blue mold disease caused by *P. expansum* is one of the major postharvest diseases in grapes [79], leading to significant economic losses. Figure 8 illustrates the biological control effect of the isolated strains against postharvest *P. expansum* in grapes. Compared to the control group, the probiotic treatment groups significantly reduced the disease incidence and decay index of *P. expansum* infection in grapes. Among the treatments, the CK group exhibited the highest disease incidence and decay index, at 91.67% and 65.0%, respectively. The mixed probiotic group was the most effective, reducing the disease incidence and decay index by 60.0% and 39.9%, respectively. The use of antagonistic microorganisms as biocontrol agents is a safe and effective strategy for addressing postharvest storage issues in fruits. LAB have shown promise as biocontrol agents for plant diseases. Previous studies by Marín et al. [22] and Chen et al. [80] demonstrated that *L. plantarum* treatment inhibit fungal growth in grapes and strawberries during storage. Additionally, both *L. plantarum* [81] and *L. rhamnosus* GG [82] have shown potential as biocontrol agents for fresh-cut fruits.

## 4. Conclusions

This study provides valuable insights into the potential of two distinct sources of LAB—human gut microbiota and fermented foods—as promising probiotic candidates. *L. plantarum* LR5-2 and *L. rhamnosus* SQ63 exhibit strong probiotic properties primarily due to their exceptional stability under gastrointestinal, bile salt, and phenol conditions, their robust adhesion to cell surfaces, and their strong antioxidant activity. Both strains are considered safe for use. In addition, LR5-2 and SQ63 demonstrated broad-spectrum antimicrobial activity, with particularly significant effects in inhibiting fungal growth. These findings highlight the potential of LAB strains as biological preservatives for grape preservation, providing an alternative to sulfur dioxide fumigation. Future research will focus on evaluating their effectiveness under extended storage conditions to identify strains that inhibit spoilage while preserving fruit quality and flavor.

## Figures and Tables

**Table 1:** The agar diffusion method was used to detect the antibacterial activity of *L. plantarum* LR5-2 and *L. rhamnosus* SQ63 against Gram-positive bacteria and Gram-negative bacteria.

Strain		Bacteriostatic Zone Diameter (mm)	
		SQ63	LR5-2
G+	*Listeria monocytogenes*	21.12 ± 0.33	22.34 ± 0.04
	*Staphylococcus aureus*	21.5 ± 0.31	22.37 ± 0.23
	*Micrococcus luteus*	26.06 ± 0.45	25.97 ± 0.27
G−	*Salmonella typhimurium*	17.06 ± 0.26	16.98 ± 0.1
	*Escherichia coli*	15.65 ± 0.37	15.19 ± 0.35
	*Pseudomonas aeruginosa*	15.46 ± 0.13	16.1 ± 0.38

**Table 2:** A double-layer plate method was used to detect the antibacterial activity of *L. plantarum* LR5-2 and *L. rhamnosus* SQ63 against mold.

Strain	Antibacterial Activity			
	SQ63		LR5-2	
*Aspergillus niger*	+	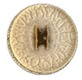	+	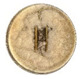
*Aspergillus flavus*	+++	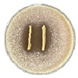	+++	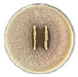
*Penicillium citrinum*	+++	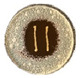	+++	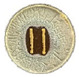
*P* *enicillium* *expansum*	+++	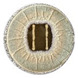	+++	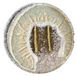
*P* *enicillium* *georgiense*	+++	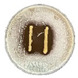	+++	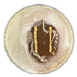

Note: ‘–’ means no inhibition and ‘+’ means weak inhibition for each lactic acid bacteria line around the total plate area of 0.1–3% of the inhibition zone; ‘++’ refers to each lactic acid bacteria line around the total plate area of 3–8% of the inhibition zone; ‘+++’ refers to each lactic acid bacteria line around the total plate area of more than 8% of the inhibition zone.

**Table 3:** Antibiotic sensitivity study of isolated strains.

Antibiotic	Antibiotic Content	Judgment Standard of Inhibition Zone Diameter			LR5-2 Drug Sensitivity Results		SQ63 Drug Sensitivity Results	
		R	I	S	Inhibition Zone Diameter	Result	Inhibition Zone Diameter	Result
	(μg/Piece)	(Resistance)	(Mediation Sensitivity)	(Sensitivity)	(mm)		(mm)	
erythromycin	15	≤13	14~22	≥23	23.82 ± 1.03	S	27.71 ± 1.04	S
penicillin	1	≤14	15~17	≥18	24.92 ± 0.37	S	31.28 ± 2.31	S
tetracycline	30	≤14	15~18	≥19	18.04 ± 0.29	I	27.25 ± 1.31	S
chloramphenicol	30	≤12	13~17	≥18	27.69 ± 1.35	S	28 ± 2.18	S
streptomycin	10	≤11	12~14	≥15	-	R	-	R
rifampicin	5	≤16	17~19	≥20	20.19 ± 0.07	S	29.78 ± 0.67	S
vancomycin	30	≤10	11~13	≥14	-	R	-	R
kanamycin	30	≤13	14~17	≥18	-	R	-	R
gentamycin	10	≤12	13~14	≥15	16.44 ± 0.96	S	16.46 ± 1.71	S
doxycycline	30	≤12	13~15	≥16	23.17 ± 0.9	S	30.64 ± 3.09	S
ampicillin	30	≤28	-	≥29	36.72 ± 0.9	S	23.08 ± 0.46	R

## Data Availability

The original contributions presented in the study are included in the article; further inquiries can be directed to the corresponding author.
